# Left ventricular twist and untwist rate provide reliable measures of ventricular function in myocardial ischemia and a wide range of hemodynamic states

**DOI:** 10.1002/phy2.110

**Published:** 2013-10-20

**Authors:** Wei Zhou, Peyman Benharash, Jonathan Ho, Yoshihiro Ko, Nikhil A Patel, Aman Mahajan

**Affiliations:** 1 Department of Anesthesiology, University of CaliforniaLos Angeles, California; 2 Department of Cardiovascular Surgery, University of CaliforniaLos Angeles, California

**Keywords:** Left ventricular twist, LV stiffness, rotation, untwisting rate

## Abstract

Although rotational parameters by speckle tracking echocardiography (STE) have been previously compared to sonomicrometry and cardiac magnetic resonance imaging, few have examined the relationship between left ventricular (LV) rotational mechanics and intraventricular measures of load-independent contractility, LV stiffness, or ventriculoarterial coupling. The aim of this study was to compare the changes in LV rotational indices to intraventricular pressure–volume (PV) relationships under a range of inotropic states induced by pharmacological interventions, acute ischemia, and changes in preload. In nine pigs, simultaneous echocardiographic imaging and LVPV measurements were performed during pharmacologically induced high or low inotropy and during acute ischemia by ligation of the left anterior descending coronary artery (LAD). Maximal ventricular elastance (E_max_), arterial elastance (E_a_), ventricular–arterial coupling (E_max_/E_a_), dP/dt, tau, and other hemodynamic parameters were determined. Dobutamine and esmolol infusions led to inversely correlated changes in hemodynamic measurements of LV function. Apical but not basal rotation and diastolic rotation rate were decreased by esmolol and increased by dobutamine. The LV twist correlates well with E_max_ (*r* = 0.83) and E_max_/E_a_ (*r* = 0.80). Apical diastolic rotation rate also correlates with dP/dt_min_ (*r* = −0.63), τ (*r* = −0.81), and LV stiffness (*r* = −0.52). LAD ligation decreased systolic and diastolic LV rotation in apical (*P* < 0.05), but not basal myocardium. Occlusion of the inferior vena cava, to reduce preload, increased apical rotation in systole and diastole. LV rotational parameters measured by STE provide quantitative and reproducible indices of global LV systolic and diastolic function during acute changes in hemodynamics.

## Introduction

The complexity of cardiac disease states treated clinically in the modern era has seen a steady increase. This can be partly attributed to the aging population and the wide array of cardiomyopathies seen in clinical practice. Despite advances in diagnostic instrumentation, accurate clinical assessment of left ventricular (LV) systolic and diastolic function remains challenging. Establishing pressure–volume (PV) relationships represents the gold standard for global assessment of LV systolic and diastolic function and yields a number of parameters describing cardiac function including end-systolic and -diastolic PV relationships (Suga et al. 1973; Sagawa et al. 1977), LV stiffness (Weiss et al. 1976), and ventricular–arterial coupling (Burkhoff et al. 2005). However, the invasiveness of PV measurements, that is, placement of an intraventricular catheter, precludes its widespread use in clinical practice.

Recent advances in speckle tracking echocardiography (STE) have facilitated the quantification of myocardial systolic and diastolic function (Helle-Valle et al. 2005; Wang et al. 2007). LV rotation is defined as the average angular displacement of all regions of myocardium relative to the center of LV cavity as seen in short axis. Fundamental to ventricular ejection, LV twist is defined as the difference between oppositely directed apical and basal rotations. While LV twist has been previously shown to correlate with systolic function (Gustafsson et al. 2009; Dalen et al. 2011; Mor-Avi et al. 2011), untwisting rate is an index of diastolic function (Burns et al. 2008, 2009).

Although STE rotational parameters have been previously compared to sonomicrometry and cardiac magnetic resonance imaging (Helle-Valle et al. 2005; Notomi et al. 2005), few have examined the relationship between LV rotational mechanics and intraventricular measures of load-independent contractility, LV stiffness, and ventriculoarterial coupling (Burns et al. 2009, 2010; Ferferieva et al. 2009; Ho et al. 2013). Whether strain or strain rate are accurate markers of intrinsic myocardial function remains controversial (Ferferieva et al. 2012).

Patients often present with derangements in systolic and diastolic function that are often not obvious using traditional measurements of LV ejection fraction (EF). Clinically, one needs to understand myocardial performance which includes systolic and diastolic functional indices, and ultimately generates cardiac output. Therefore, a more intricate assessment of cardiac function that more closely mirrors invasive measurements with an LV volumetric catheter is needed. The aim of this study was to compare the changes in LV rotational indices to intraventricular pressure–volume relationships under a range of inotropic states induced by pharmacological interventions, acute ischemia, and changes in preload. We chose to record both load-independent and dependent parameters as they pertain to basic physiology and clinical practice, respectively.

## Methods

### Animal preparation

The animal study was approved by the Chancellor's Animal Research Committee at University of California, Los Angeles and animals were treated in compliance with the National Institutes of Health guidelines for the care and use of laboratory animals. Nine Yorkshire pigs weighing 40–45 kg were anesthetized intramuscularly with ketamine (15–25 mg/kg) and xylazine (2 mg/kg) and mechanically ventilated via endotracheal intubation. Anesthesia was maintained by inhaled isoflurane (1–2%) and intermittent intravenous boluses of fentanyl (5–10 μg/kg). The electrocardiogram was monitored from limb leads. The femoral artery and jugular vein were cannulated for blood pressure measurement and drug infusion. The animals underwent median sternotomy, and a snare was placed around the inferior vena cava (IVC) for preload manipulation. Occluding snares were placed around the left anterior descending (LAD) coronary artery.

### Invasive hemodynamic measurements

A 5F pigtail 12-pole multielectrode combination conductance-pressure catheter (Millar Instruments, Inc., Houston, TX) was placed in the left ventricle (LV) via the right carotid artery and connected to a conductance processor (MPVS Ultra, Houston, TX) for continuous measurement of LV pressure and volume (Baan et al. 1984). Proper electrode position was confirmed by ensuring that volume waveforms were obtained from each individual pair of electrodes on the catheter. LV volume was calculated by injecting a hypertonic saline (0.2 g NaCl/mL, 2 mL) into the right atrium to obtain a constant offset volume (Vc). We measured Vc before and at the end of each experiment and confirmed that Vc remained stable. The conductance catheter method of measuring LV volume was described in detail in our previous studies (Syuu et al. 2001, 2003) and elsewhere (Baan et al. 1992).

Hemodynamic indices were obtained from steady-state PV loops during sinus rhythm, and PV relationships were derived during serial preload reduction by IVC occlusion, as previously reported (Syuu et al. 2003; Burkhoff et al. 2005). LV systolic function was assessed by EF, end-systolic pressure, maximum rate of pressure change (dP/dt_max_), dP/dt_max_/EDV, a sensitive isovolumic phase measure of LV contractility (Kass et al. 1989), and E_max_, the slope of the end-systolic PV relationship (Suga et al. 1973). As a measure of afterload, arterial elastance (E_a_) was calculated as ESP divided by stroke volume (SV) (Sunagawa et al. 1985). The ratio of E_max_ to E_a_ was determined to express the balance between ventricular contractility and arterial resistance (ventricular–arterial coupling). Diastolic function was assessed by the LV end-diastolic pressure (LVEDP), isovolumetric relaxation time constant (τ), and the minimum rate of LV pressure change (dP/dt_min_) (Weiss et al. 1976; Zile et al. 2004). LV chamber stiffness (dP/dV) in diastole was calculated as the slope during the filling phase of a single PV loop, averaged over five beats. The PV relationship during the filling (from the start of LV filling to the end of diastole) was fit to the exponential function LVEDP = *c*·exp (*β*·LVEDV), where *c* is the diastolic PV coefficient, *β* is the chamber stiffness constant, and LVEDV is LV end-diastolic volume. The parameters *β* and *c* represent load-independent indices of diastolic function (Garcia et al. 2001; Kasner et al. 2007; Burns et al. 2009).

### STE recording and analysis

Open-chest epicardial echocardiography was performed using a GE Vivid 7 Dimension system (GE Vingmed Ultrasound, Horten, Norway) with a 10 MHz transducer. Image quality was improved using a 5 × 3 cm section of preharvested pig liver affixed to the echo probe with parafilm. Echocardiographic and hemodynamic data were acquired sequentially for each manipulation, while assuring similar hemodynamics between the two. A phase array transducer (GE 10S) with frequency of 10 MHZ, frame rate of 70 per sec, and sector widths manually adjusted to optimize speckle quality, was used. Three cardiac cycles were recorded for subsequent offline analysis. LV rotation measurements were obtained using speckle tracking software (EchoPAC PC version 8.0, GE Vingmed Ultrasound) at the basal (identified by the mitral valve) and apical (at the level when papillary muscles just disappear) levels. Counterclockwise rotation was defined as a positive angle and clockwise rotation as a negative angle when viewed from the apex toward the base. The accuracy of the image tracking software was verified manually.

### Experimental protocol

The animals were stabilized for a 30-min period prior to baseline measurements. Hemodynamic and STE images were recorded at baseline and 30–35% reduction in end-diastolic volume by 1 min of IVC occlusion. A 30–35% decrease in preload was confirmed by the Millar catheter measurement of LVEDV. LV systolic and diastolic function were examined at baseline in addition to manipulated inotropic states: low inotropic state (esmolol, 100–200 μg/kg/min) and high inotropic state (dobutamine, 10–15 μg/kg/min), titrated to a ∼20% change in dP/dt_max_. Hemodynamic parameters were allowed to stabilize for ∼15 min at each dose prior to data acquisition; following cessation of infusion of each drug, hemodynamic values were allowed to return to baseline prior to starting the next infusion. Following drug infusion hemodynamic parameters were allowed to return to baseline values, at which point, the LAD was then occluded for 30 min. Sequential STE images and PV measurements were acquired repeatedly during end-expiratory apnea on a mechanical ventilator. Data acquisition was repeated three times and averaged for each condition with >3-min intervals between measures. All animals were euthanized at the end of the study with pentobarbital (100 mg/kg) and KCl solution intravenously.

### Statistical analysis

Analysis was performed using Sigma Stat (Ver.3.1, San Jose, CA) and data are presented as mean ± SD. The hemodynamic and echocardiographic measurements were compared in the various experimental stages by use of repeated analysis of variance followed by the post hoc Bonferroni correction. Linear regression analyses were performed to determine relationships between echocardiographic and PV loop diastolic indices. Correlations between echocardiographic and PV parameters were determined using the Pearson correlation coefficient. Nonlinear terms were considered and included only if significant. Statistical differences were considered significant at *P* < 0.05.

#### Inter- and intraobserver reproducibility

Two independent observers processed echocardiography images for apical rotation in all animals. Each observer analyzed the same image two times. Intraobserver variability was determined by having one observer repeat the measures 1 month after the initial analysis. Interobserver and intraobserver variability was assessed using intraclass correlation coefficient (ICC) with 95% confidence intervals as we demonstrated previously (Zhou et al. 2013).

## Results

### STE versus PV loop analysis

**Figure 1:**
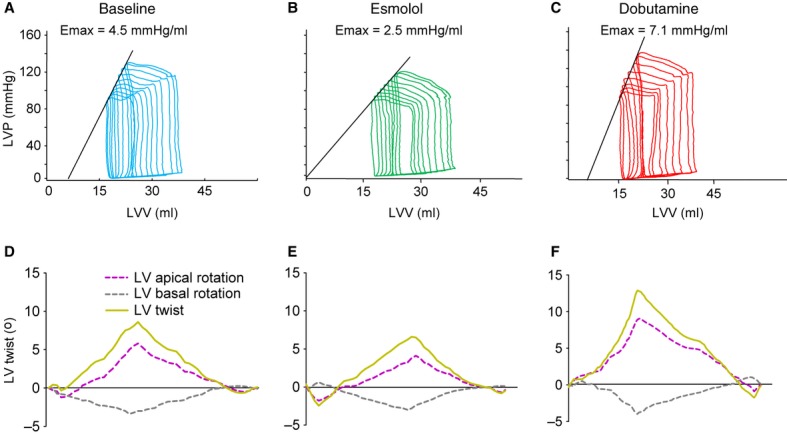
Pressure–volume loops and STE parameters. (A–C) Typical pressure–volume loops were recorded from one animal under baseline conditions (left) and during infusion of esmolol (middle) and dobutamine (right). The E_max_ was calculated as the linear slope of end-systolic points from multiple loops obtained during IVC occlusion. E_max_ decreased with esmolol and increased with dobutamine. Two-dimensional STE was performed simultaneously and STE parameters were determined off line. (D–F) left ventricular twist is decreased from baseline with esmolol and increased with dobutamine.

Figure 1 shows representative PV loops, LV apical basal rotation, and LV twist at baseline, and during infusion of dobutamine (β1 receptor stimulation) and esmolol (β receptor antagonism). Esmolol decreased while dobutamine increased E_max_ from baseline values (Fig. 1 A–C). These changes in PV values were mirrored by changes in STE parameters. Global apical rotation decreased in the presence of esmolol and increased with dobutamine (Fig. 1 D–F).

Table 1 summarizes changes in systolic and diastolic parameters for PV and STE data. As expected, systolic and diastolic function varied with low and high inotropy. LV stiffness parameters also varied with inotropic state. The LV chamber stiffness constant (*β*) varied inversely with inotropic state measuring 0.061 ± 0.017 and 0.023 ± 0.011 during low and high inotropic states. E_max_/E_a_ also significantly varied with low and high inotropy (0.66 ± 0.11 vs. 1.96 ± 0.22).

**Table 1:** Hemodynamic and twist response to pharmacological interventions and myocardial ischemia

	LV systolic and diastolic function			Acute ischemia	
	
	Baseline	Esmolol	Dobutamine	Preischemia	Ischemia
Invasive					
Heart rate, bpm	75 ± 9	65 ± 8^1^	92 ± 10^1^	78 ± 7	87 ± 8^2^
End-systolic pressure, mmHg	101 ± 11	89 ± 8^1^	120 ± 12^1^	102 ± 11	84 ± 11^2^
End-diastolic pressure, mmHg	5 ± 2	7 ± 1	7 ± 2	6 ± 1	12 ± 3^2^
Stroke volume, mL	17 ± 2	15 ± 2^1^	22 ± 3^1^	19 ± 3	14 ± 3^2^
dP/dt_max_, mmHg/sec	3407 ± 507	2247 ± 440^1^	5055 ± 735^1^	3508 ± 585	1740 ± 410^2^
dP/dt_max_/EDV, mmHg/sec/mL	90 ± 10	64 ± 9^1^	165 ± 11^1^	97 ± 9	42 ± 7^2^
E_max_, mmHg/mL	6.3 ± 0.8	3.9 ± 0.6^1^	10.8 ± 1.2^1^	6.0 ± 1.0	2.4 ± 0.5^2^
E_a_, mmHg/mL	5.9 ± 1.1	5.9 ± 0.8	5.5 ± 0.7^1^	5.4 ± 0.9	6.0 ± 1.0
E_max_/E_a_	1.06 ± 0.16	0.66 ± 0.11^1^	1.96 ± 0.22^1^	1.11 ± 0.14	0.40 ± 0.10^2^
Tau, msec	30 ± 5	35 ± 6^1^	23 ± 6^1^	28 ± 7	39 ± 9^2^
dP/dt min, mmHg/sec	−2651 ± 552	−2121 ± 482^1^	−3843 ± 453^1^	−2807 ± 501	−2061 ± 421^2^
b	0.23 ± 0.07	0.38 ± 0.11^1^	0.16 ± 0.04^1^	0.24 ± 0.08	0.41 ± 0.10^2^
c	0.77 ± 0.21	0.57 ± 0.13^1^	1.37 ± 0.35^1^	0.80 ± 0.25	0.67 ± 0.26
β	0.047 ± 0.015	0.061 ± 0.017^1^	0.023 ± 0.011^1^	0.042 ± 0.012	0.059 ± 0.016^2^
2D speckle tracking echocardiography					
Apical rotation, °	6.2 ± 1.1	4.1 ± 0.8^1^	8.5 ± 1.7^1^	5.3 ± 1.2	2.2 ± 0.6^2^
Basal rotation, °	−3.3 ± 0.5	−2.8 ± 0.4	−3.7 ± 0.6	−3.2 ± 0.3	−2.9 ± 0.5
LV twist, °	9.5 ± 1.6	6.9 ± 1.1^1^	12.2 ± 1.8^1^	8.5 ± 1.3	5.1 ± 1.0^2^
Apical diastolic rotation rate, °/sec	−41 ± 6	−27 ± 6^1^	−63 ± 7^1^	−36 ± 5	−24 ± 4^2^
Basal diastolic rotation Rate, °/sec	36 ± 5	32 ± 4^1^	42 ± 7	28 ± 4	30 ± 3

Values are mean ± SD. b, LV stiffness; c, curve-fitting constant; β, stiffness constant.

1 *P* < 0.05 versus mid.

2 *P* < 0.05 ischemia versus pre-ischemia.

Apical but not basal rotation and diastolic rotation rate were decreased by esmolol and increased by dobutamine suggesting that apical and basal rotation responded differently to pharmacological changes in inotropy.

**Figure 2:**
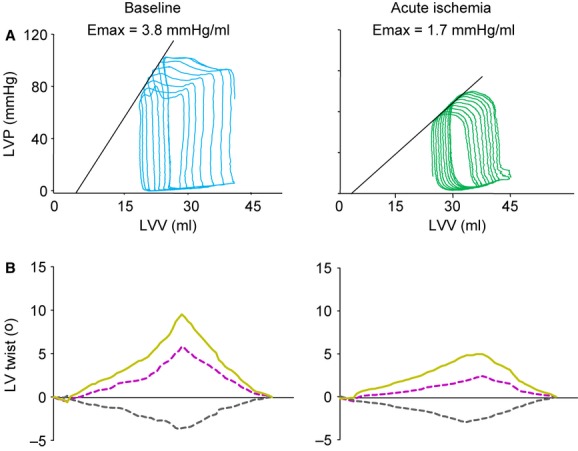
Pressure–volume Loops and STE parameters during acute ischemia. (A) E_max_ decreased during ischemia. Simultaneous STE measurements show that the peak apical rotation (B) decreased during acute ischemia by LAD occlusion.

Acute ischemia due to LAD occlusion decreased both systolic and diastolic function and increased LV stiffness (Table 1). E_max_ decreased from 6.0 ± 0.8 at baseline to 2.4 ± 0.5 mmHg/mL during acute ischemia, while relaxation τ increased from 28 ± 7 to 39 ± 9 msec. During LAD occlusion, apical rotation decreased, while basal rotation did not change (Fig. 2). LAD occlusion decreased apical, but not basal, diastolic rotation rate. Changes in STE parameters during acute ischemia were consistent with observations in PV data analysis.

### Correlations between STE and PV loop parameters

**Figure 3:**
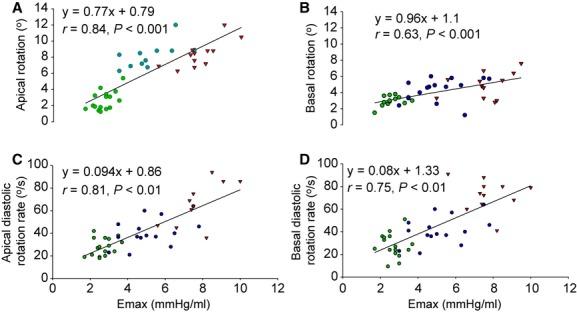
Correlations between LV rotational parameters and E_max_. Inotropic state was varied pharmacologically using esmolol and dobutamine at varied doses. Low inotropic states (•) were induced using high esmolol concentrations; high inotropic states (▼) were induced with high dobutamine concentrations; and baseline (○). Lines show the linear regressions fitted from pooled data from all animals. Apical STE parameters (A, C) had better correlations with E_max_ than corresponding basal STE parameters (B, D).

To validate the accuracy of STE measurements in response to changes in inotropy, we correlated STE with PV parameters. The correlation coefficients between STE and PV loop systolic and diastolic parameters are summarized in Tables 2 and 3. Both LV rotation and twist correlate well with invasive indices of systolic function: dP/dt_max_, dP/dt_max_/EDV, EF, ESP, and E_max_. Apical rotation correlates better with E_max_ than basal rotation (Fig. 3A, *r* = 0.84 vs. 0.63). Notably, STE diastolic parameters for apical and basal diastolic rotation rate also correlate well with E_max_ (Fig. 3C, *r* = 0.81 and 0.65, respectively).

**Table 2:** Correlation of LV twist and rotation with invasive systolic indexes

	ESP	dP/dt_max_	dP/dt_max_/EDV	E_max_/E_a_
Apical Rotation				
*r*	0.73	0.79	0.83	0.79
*P*	0.001	0.001	0.001	0.001
Basal rotation				
*r*	0.50	0.50	0.58	0.65
*P*	0.001	0.001	0.002	0.001
LV twist				
*r*	0.72	0.80	0.85	0.80
*P*	0.001	0.002	0.002	0.001

**Table 3:** Correlation of LV diastolic rotation rate with invasive diastolic indexes

	dP/dt_min_	EDP	b	c
Apical diastolic rotation rate				
*r*	−0.63	−0.62	−0.52	0.43
*P*	0.001	0.001	0.001	0.01
Basal diastolic rotation rate				
*r*	−0.48	−0.42	−0.45	0.39
*P*	0.004	0.01	0.009	0.02

**Figure 4:**
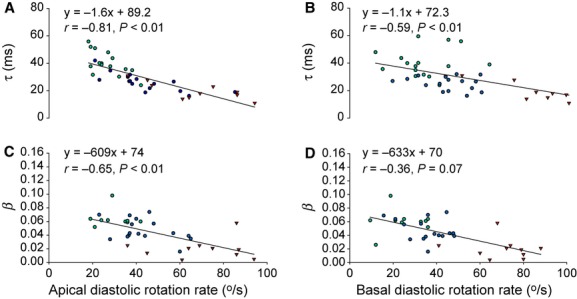
Correlations between LV diastolic rotation rate and invasive diastolic parameters. (A, B) Time constant of LV relaxation (τ) closely correlates with apical and basal LV diastolic rotation rate. (C, D) LV chamber stiffness (β) correlates with apical but not basal LV diastolic rotation rate.

We then examined correlations between PV and STE diastolic parameters (Fig. 4, Table 3). Relaxation time constant correlated well with apical and basal diastolic rotation rate (*r* = −0.81 and −0.59, respectively). The LV chamber stiffness constant (*β*) correlated better with apical than basal diastolic rotation rate (−0.65 vs. −0.36). Thus, apical LV diastolic rotation rate provided good surrogate measures of diastolic function, as demonstrated by good correlation with diastolic PV parameters.

### Effect of IVC occlusion on LV twist and untwist

To determine whether STE parameters were affected by changes in preload, we measured PV loops and STE values during IVC occlusion. A 30–35% reduction in preload by IVC occlusion (Table 4) predictably decreased ESV (20%), EDV (29%), ESP (19%), SV (33%), and dP/dt_max_ (15%) but increased dP/dt_max_/EDV (18%); however, heart rate (HR), EDP, and relaxation time constant (τ) remained unchanged. During IVC occlusion, apical rotation significantly increased (23%), and time to peak apical rotation shortened (14%). The magnitude of apical diastolic rotation rate also increased (23%) during IVC occlusion.

**Table 4:** Hemodynamic and STE parameters during inferior vena cava occlusion

	Relative change LV end-diastolic volume %	

	Preocclusion	30–35
Invasive		
Heart rate, bpm	79 ± 12	82 ± 11
End-systolic pressure, mmHg	98 ± 9	82 ± 8^1^
End-diastolic pressure, mmHg	7 ± 2	5 ± 2
Stroke volume, mL	18 ± 2	12 ± 3^1^
dP/dt_max_, mmHg/sec	3080 ± 550	2630 ± 503^1^
dP/dt_max_/EDV, mmHg/sec mL^−1^	81 ± 11	96 ± 10^1^
Tau, msec	33 ± 6	36 ± 5
b	0.21 ± 0.02	0.19 ± 0.03
c	0.81 ± 0.23	0.83 ± 0.25
β	0.045 ± 0.013	0.048 ± 0.013
2D speckle tracking echocardiography		
Apical rotation, °	5.8 ± 2.1	7.1 ± 2.9^1^
Basal rotation, °	−2.8 ± 0.6	−3.0 ± 0.7
LV twist, °	8.6 ± 1.5	10.1 ± 2.3^1^
Apical diastolic rotation rate, °/sec	−68 ± 13	−83 ± 12^1^
Basal diastolic rotation rate, °/sec	35 ± 6	38 ± 8

Values are mean ± SD.

1 *P* < 0.05 versus Preocclusion.

We checked the reproducibility of apical rotation. The ICC for intraobserver variability was 0.92 while for interobserver variability was 0.86.

## Discussion

In this study, we demonstrate the validity of STE in determining cardiac functional parameters during pharmacological inotropic manipulation, acute regional cardiac ischemia, and acute preload changes. Several novel insights can be learned from this study. Although LV twist has been shown to be an accurate measure of systolic function, we have now shown that it also correlates well with load-independent indices of ventricular contractility such as E_max_ and dP/dt_max_/EDV as well as ventricular–arterial coupling such as E_max_/E_a_. LV twist is also a sensitive parameter of systolic function during preload manipulation. Second, although previously correlated with isovolumic relaxation time constant (τ) (Burns et al. 2009), LV diastolic rotation rate also correlates well with other invasive indices, including, dP/dt_min_, and LV chamber stiffness (*β*), the last of which has remained controversial. Thus, LV diastolic rotation rate serves as a good surrogate measure of diastolic function. Although systole and diastole are likely coupled and each rotational parameter does not specifically indicate function during each phase alone, given the close correlation of LV twist and untwist with E_max_, we suggest that the STE rotational parameters may be the most accurate noninvasive measures of global myocardial function.

### Systolic function and LV twist

Speckle tracking analysis provides angle-independent information on motion and deformation of LV, which is a major improvement over standard echocardiographic approaches (Helle-Valle et al. 2005). LV twist is predominantly counterclockwise at the apex and clockwise at the base during systole (Helle-Valle et al. 2005; Notomi et al. 2005). Our results show that basal and apical myocardium responds differently to pharmacological alterations within a range of physiological inotropic states. Apical rotation but not basal rotation was decreased by esmolol and increased by dobutamine. The apicobasal differences in rotational response may arise from regional differences in myofibril architecture (Greenbaum et al. 1981), or may suggest differential local densities of adrenergic receptors or nerve terminals (Oh et al. 2006). Consistent with a previous study (Nagel et al. 2000), we found that 30 min of LAD occlusion decreased apical rotation but not basal rotation, suggesting focal LV dysfunction in the apical region. While circumferential strain in basal and apical regions may reflect regional systolic motion (measured but not reported here), LV twist which is calculated as the difference between apical and basal rotations, correlates well with E_max_ and dP/dt_max_/EDV, suggesting that LV twist may serve as a sensitive marker of global systolic function.

### Diastolic function and LV diastolic rotation rate

LV diastolic rotation rate may serve as a critical determinant of early diastolic function (Rademakers et al. 1992; Notomi et al. 2005; Burns et al. 2009). Commensurate with the literature (Wang et al. 2007), we found that LV diastolic rotation rate correlates well with the isovolumic relaxation time constant (τ). Our data also show that LV diastolic rotation rate correlates with LV stiffness parameters which are established parameters of ventricular diastolic function. A previous report has suggested that LV diastolic rotation parameters are not related to LV chamber stiffness (Burns et al. 2008). In this previous study, the inotropic variability was maintained within a limited range, unlike in the present study where esmolol and dobutamine were applied to provide a much wide range of cardiac contractility. Thus, the apparent discrepancy in diastolic rotation rate–stiffness correlation might be due to the differences in the dynamic ranges of inotropy during which invasive PV parameters were obtained. Furthermore, the previous study used recoil and recoil rate instead of diastolic rotation rate as defined and used in the present study.

One of the novel findings of our study is that apical diastolic rotation rate closely correlates with E_max_, an excellent load-independent marker of contractility. Previous work has noted that peak untwisting velocity correlated well with other STE parameters such as systolic peak rotation, positive rotation rate, and peak positive rotation velocity (Burns et al. 2008). To our knowledge, this is the first demonstration of a robust relationship between LV diastolic rotation rate and load-independent contractility, E_max_. Systolic twist and diastolic untwist parameters both correlated well with E_max_, which represents maximal tissue elastance, suggesting that LV twist might form a mechanistic link between the systolic and diastolic phases of the cardiac cycle.

Altered ventricular diastolic rotation rate may play a critical role in abnormal diastolic function. Clinical studies show that LV diastolic rotation rate is decreased in magnitude and rapidity under conditions of aortic stenosis (Stuber et al. 1999), hypertrophic cardiomyopathy (Maier et al. 1992), and severe LV hypertrophy (Takeuchi et al. 2007). The present findings provide the systematic evidence to demonstrate that LV diastolic rotation rate, as determined by STE, is an accurate marker for evaluation of diastolic function, as shown by good correlation with PV parameters of relaxation and stiffness. Therefore, LV diastolic rotation rate may prove valuable as a diagnostic tool for disease stratification and for quantification of diastolic function.

### Load dependence and LV twist and untwisting rate

Load dependence of ventricular twist has been controversial. A previous study showed that LV twist is not affected by changes in preload or afterload (Hansen et al. 1991). Studies in transplanted human hearts have suggested that LV twist was not affected by pressure and volume loading (Moon et al. 1994). On the other hand, one animal study showed that twist, measured optically, was primarily a function of volume and was dependent on both preload and afterload (Gibbons Kroeker et al. 130-141[Bibr b11]). In an isolated working heart model, LV twist increased at higher EDV when ESV was held constant, and decreased at higher ESV when EDV was held constant (Hansen et al. 1991).

During IVC occlusion, we observed that apical rotation and diastolic rotation rate significantly increased while basal rotation and diastolic rotation rate remained the same. Both ESV and EDV decreased during IVC occlusion. LV twist and ESV have been observed to be determinants of LV diastolic rotation rate (Wang et al. 2007), which is consistent with our findings in the apical, but not basal cardiac regions. Indeed, changes in preload affect twist dynamics. Greater twist/untwist occurs during isovolumic periods, and maximum apical twist is reached earlier in systole with low preload. Therefore, LV twist is enhanced by conditions associated with smaller end-systolic volumes.

The LV untwisting occurs primarily during isovolumic relaxation, before filling begins, and is related to the elastic restoring forces that have been reported to be a function of LV systolic volume (Wang et al. 2007). Thus, it is not surprising that apical rotation and diastolic rotation rate were both increased during decreased preload associated with IVC occlusion. Enhanced LV twist during IVC occlusion is potentially explained by reflex sympathetic activation. However, others have observed the opposite finding: that is, increasing preload increase apical rotation. This discrepancy may be accounted for by the time period of preload change which in our case was brief and in cases of Park et al. (2010) and Weiner et al. (2010) was prolonged. We also observed an increase in dp/dt_max_ normalized with EDV to reduce the preload effect during IVC occlusion, indicating that LV twist may serve as a sensitive parameter of systolic function during preload manipulation. Others such as Opdahl et al. (2012) have reached conflicting conclusions and have demonstrated that LV diastolic rotation rate is independent of preload and chamber ventricular relaxation. Such differences may be attributable to the animal models utilized and the depth of anesthesia during the experiments (Opdahl et al. 2012). We have recently demonstrated a strong relationship between STE derived LV apical rotation with LV function determined by invasive hemodynamic parameters during pharmacologic afterload manipulation (Ho et al. 2013). LV apical rotation is sensitive to the development of afterload-induced impairment of LV function in systole and diastole. Taken together, the STE parameters reflect changes in ventricular function and may serve as reliable indices of load-dependent changes in cardiac function.

### Clinical implications

Assessment of LV systolic and diastolic pump properties is fundamental to advancing the understanding of cardiovascular pathophysiology and therapeutics. Despite widespread use of echocardiography to evaluate systolic and diastolic function, conventional echo parameters, such as EF and early atrial wave Doppler ratios are limited in accuracy and practicality. Recent developments in STE have received much attention due to its clinical availability and feasibility. In the present study, we have demonstrated that the LV twist and untwist correlate well with PV measurements of both systolic and diastolic function. Similar demonstrations of the utility of STE have been made using sonomicrometry. However, the techniques of sonomicrometry and conductance catheter are of limited clinical value. We feel that STE rotational parameters may serve as reliable clinical and research tools for assessment of heart function and may provide insights into cardiovascular diseases and the development of new therapies. Unlike invasively measured parameters during isovolumic contraction and relaxation, STE rotational parameters, in our opinion, reflect overall myocardial performance and indeed may be more valuable in clinical applications.

### Study limitations

Being a three-dimensional structure, it is likely that rotational parameters are also related to longitudinal and circumferential strains. Others have reported on such interdependence previously (Meimoun et al. 2011). In order to obtain high-quality images and reduce motion artifact during acquisition, we focused on twist, which can be obtained by temporal comparison of two short-axis slices. These sections might be simpler to acquire during clinical work.

The image quality of the recordings must be high to achieve correct tracking, which requires proper adjustment of frame rate, probe frequency, and focal plane. In the present study, tracking quality was evaluated visually. Regions of poor tracking were replaced by areas of better speckle quality or disregarded. One problem with STE in LV short-axis images is that longitudinal motion causes the myocardium to move in and out of the image plane. As a consequence, speckles generated from the ultrasound beam could represent myocardial tissues from different cross-sectional levels during the cardiac cycle. This problem is most pronounced at the LV base (positioned furthest from the echo probe), whereas the images in the LV apex remained relatively stationary. In our study, the relationships between STE and PV loop parameters were better correlated at the apical level than at the base. The observed apicobasal differences in rotation are consistent with the known mechanical properties of the heart; however, possible motion artifacts cannot be completely excluded and would likely have been greater in basal rather than apical recordings.

As this study was performed in accordance with National Institutes of Health Guidelines for the Care and Use of Laboratory Animals, anesthesia was maintained to abolish the pain response at all times. The difficulty in assessing the depth of anesthesia is ubiquitous in studies of anesthetized animals and may affect the cardiovascular response to various hemodynamic manipulations.

## Conclusions

The present findings demonstrate that STE parameters correlate well with PV data under a wide range of cardiac inotropic states. LV twist and untwisting rates assessed by STE provide quantitative and reproducible indices of global LV systolic and diastolic function during acute hemodynamic changes. Thus, STE rotational parameters may serve as flexible clinical and research tools for assessment of heart function.
